# Investigating the potential of novel non-woven fabrics for efficient pollination control in plant breeding

**DOI:** 10.1371/journal.pone.0204728

**Published:** 2018-09-28

**Authors:** John C. Clifton-Brown, Hannah Senior, Sarah J. Purdy, Richard Horsnell, Bernd Lankamp, Ann-Katrin Müennekhoff, Daljit Virk, Estelle Guillemois, Vera Chetty, Alan Cookson, Sarah Girdwood, Gabi Clifton-Brown, Mei Lie MC Tan, Danny Awty-Carroll, Alison R. Bentley

**Affiliations:** 1 Institute of Biological, Environmental and Rural Sciences, Plas Gogerddan, Aberystwyth University, Aberystwyth, United Kingdom; 2 PBS International, Scarborough, United Kingdom; 3 The John Bingham Laboratory, NIAB, Cambridge, United Kingdom; 4 KWS SAAT SE, Einbeck, Germany; 5 Nonwovens Innovation & Research Institute Ltd, Leeds, United Kingdom; 6 CreaNova Consultancy, Hann-Münden, Germany; Banaras Hindu University, INDIA

## Abstract

Plant breeding is achieved through the controlled self- or cross-pollination of individuals and typically involves isolation of floral parts from selected parental plants. Paper, cellulose or synthetic materials are used to avoid self pollination or cross contamination. Low seed set limits the rate of breeding progress and increases costs. We hypothesized that a novel ‘non-woven’ fabric optimal for both pollination and seed set in multiple plant species could be developed. After determining the baseline pollen characteristics and usage requirements we established iterative three phase development and biological testing. This determined (1) that white fabric gave superior seed return and informed the (2) development of three non-woven materials using different fibre and layering techniques. We tested their performance in selfing and hybridisation experiments recording differences in performance by material type within species. Finally we (3) developed further advanced fabrics with increased air permeability and tested biological performance. An interaction between material type and species was observed and environmental decoupling investigated, showing that the non-woven fabrics had superior water vapour transmission and temperature regulation compared to controls. Overall, non-woven fabrics outperformed existing materials for both pollination and seed set and we found that different materials can optimize species-specific, rather than species-generic performance.

## Introduction

Pollination control bags (PCBs) are widely used in plant breeding allowing the precise and controlled selfing or intercrossing of individuals. However, despite their importance, they can inherently limit the rate, and increase the cost, of breeding progress due to restriction of light required for biological processes, elevation of temperatures unfavourable for pollen production, release and fertilization, and in the creation of adverse microclimates supporting pest and disease damage. This has been previously shown to reduce seed yield and seed weight for first stage trialing [1] and to give false negatives on both self and cross compatibility [2], a crucial component in progeny evaluation trials [3] for crops such as sugar beet.

The quality and quantity of light transmitted through a PCB to the reproductive plant parts impacts pollination. Pollen tube elongation has been shown to be influenced by light spectral quality [4], with pollen tube length shown to be maximised by red light and minimised by blue light. Exposure to far-red light also decreased pollen tube length although could be counteracted by treatment with red light. This suggests that the ratio of red: far-red light is an important factor in pollen tube extension and indicates that light penetration and light filtering are important factors in PCB design, particularly in cases where plants are bagged prior to the onset of reproductive development. Therefore, coloured materials that adjust light wavelengths internally to increase the red:far red ratio may improve pollination, and similarly a PCB that adjusted light penetration towards the blue end of the spectrum might reduce pollination. Further, available light spectrum can influence plant morphology and seed yield. Yields have been found to be highest under white light compared to (in descending order) red light with 10% blue light, red light with 1% blue light and red light only [5]. The colour of the PCB could therefore influence pollen growth and subsequent seed yield if, for example, a bag that reflected light in the red region of the spectrum but allowed penetration by blue light was used [5].

Temperature is also a key consideration in PCB design and there is evidence that high temperatures in general reduce seed set. In sorghum it was found that temperatures over 32°C negatively impacted seed production [6]. In temperate grasses, such as wheat, recommended day/night temperatures are significantly lower, with reports of maximum kernel weight achieved at 15/10°C [7]. In the model monocot *Brachypodium distacyon* temperature increases from 24 to 28°C have been shown to reduce grain dry weight per plant by ~50% and a further increase to 32°C eliminated seed set completely [8]. Elevated in-bag temperatures have been shown to have a negative impact on pollen viability [7–8]. As PCBs cover the reproductive plant parts, their impact on temperature is particularly important. Previous work has shown that during periods of high irradiance PCBs made from transparent materials can have internal temperate that are 6 to 10°C higher than ambient [9–10].

Linked to temperature, high humidity can also reduce pollination and seed set through the creation of an unfavourable reproductive micro-climate or a favourable environment for pests and diseases. There is empirical evidence that seed set is lowered by dry air and that low humidity is associated with a reduction in pollen lifespan and viability. In *Lolium*, a high humidity treatment reduced seed set by 60% [2]. In contrast, in sorghum crossing experiments [10] a trend towards greater seed yield was observed in the higher humidity treatment. High levels of humidity however support the colonization and growth of pests and pathogens [11].

Aside from modification of the reproductive environment, cost and usability are key factors in the adoption of specific PCBs in breeding programmes. Standard PCBs made from paper (including glassine) and cellulose are cheap but are easily damaged by birds [12], insects [13], wind [14], deliberate daily bag shaking for pollen dispersion, water, and diseases [15] or slugs. They can also be difficult to support when multi-stem bundles of panicles are enclosed. Usability issues are magnified depending on the crossing environment, with the greatest potential damage in field-based breeding operations. Therefore, more robust PCBs made from tougher non-woven fabrics [16] have potential practical and economic advantages for field-based breeders. The potential for economic benefit in a field- or glasshouse-based breeding programme is based on an estimated cost saving per cross from the use of superior materials. We estimate, based on generalized costs from the testing used in this study, that a PCB represents 1–6% of the total cost (lighting, space, labour, consumables, overheads) per cross, dwarfed by the cost of labour and greenhouse space. Therefore, we propose that improving the performance of the PCB, itself a minor cost, will reduce the total number of crosses required per breeding programme, delivering substantial labour and space reductions, and therefore accruing economic gain.

In this study we hypothesized that a single novel breathable non-woven fabric optimal for both pollination and seed set in multiple plant species could be developed. We manufactured, for the first time, new non-woven materials for specific functional use in PCBs and conducted detailed assessment of their performance. We developed an iterative three phase development and biological testing pipeline across multiple plant species. Experiments characterized physical properties and biological performance with Arabidopsis, Miscanthus, wheat and sugar beet. We hypothesized (1) that selective spectral light filtering from different bag colours could alter seed production. We also hypothesized (2) that PCBs made from novel non-woven fabrics with synthetic and natural fibres could provide a generic material for PCBs for multiple crop types with associated benefits of standardization and ease of industrial upscaling. We then (3) manufactured non-woven fabrics with increased light transmission and breathability, hypothesizing that these parameters have a major impact on seed production. We concurrently developed a series of physical tests on the new PCB materials in order to understand the complexities of species and environmental interactions.

## Materials & methods

### Plant material

Four different plant species were selected for PCB biological testing. They were the model dicot Arabidopsis (*Arabidopsis thaliana;* self pollinated), the bioenergy grass Miscanthus (*Miscanthus sinensis*; wind pollinated) and the arable field crops wheat (*Triticum aestivum*; self pollinated) and sugar beet (*Beta vulgaris*; wind pollinated). These species were selected to represent a variety of breeding systems (all species are bisexual but their breeding system differs), PCB uses (plant part and duration of use) and pollen sizes (Table 1). In order to evaluate their pollen characteristics, specimens of pollen or anthers were assessed using scanning electron microscopy (SEM). Pollen samples from Arabidopsis, wheat and sugar beet were prepared for SEM by mounting undehydrated/unfixed pollen grains onto 15 mm aluminium SEM stubs using double-sided adhesive conductive carbon disks (Agar Scientific, Stansted, UK). Miscanthus anthers were fixed using 2.5% glutaraldehyde in 0.1M sodium cacodylate then stained using 1% osmium tetroxide solution in sodium cacodylate and attached via adhesive disks to SEM stubs, as above. All stubs were coated with gold for 5 minutes using a Polaron E5000 sputter coater and imaged using a Hitachi S-4700 field emission SEM, using the Ultra High Resolution mode at accelerating voltages of 5 or 10kV. Images were captured at a resolution of 2560x1920.

**Table 1:** Details of the four selected plant species used in the biological testing of a range of novel and control pollination control bags in Phase II and III.

Name (as used herein)	Arabidopsis	Miscanthus	Wheat	Sugar beet
Family	Cruciferae	Poaceae	Poaceae	Amaranthaceae
Species used in tests	*Arabidopsis thaliana*	*Miscanthus sinenesis*	*Triticum aestivum*	*Beta vulgaris*
System tested	Self-pollination	Cross-pollination	Cross-pollination	Self-pollination
Parental lines used in biological testing	Columbia “Col-0”	Parent A, EMI-11 (MS88-110. Parent B, ADAS_PN95/20	Nine elite wheat cultivars ‘Alchemy’, ‘Brompton’, ‘Claire’, ‘Hereward’, ‘Paragon’, ‘Rialto’, ‘Robigus’, ‘Soissons’ and ‘Xi-19’	Phase II: G_01, G_98; Phase III: eight genotypes denoted 1–8.
Pollen size (μm length x width)	30 x 15	25 x 30	50 x 60	15 x 20
PCB enclosed plant parts	Whole plant	Whole panicles, usually including the flag leaf	Single ear	Whole plant
Duration of enclosure in PCBs	From flowering stem initiation	From panicle exertion until seed set	Emasculation to seed set	From initiation of flowering

### Phase I PCB colour testing

In order to determine the optimal colour for the novel PCBs, polypropylene (PP) spunbond bags (40cm length x 20cm width; 0.52mm thickness; 70gm^-2^ weight) were produced in seven colours (blue, brown, white, yellow, red, black and green) and physically assessed using a spectrophotometer to compare light transmittance across 5nm wavelength gradations from 350 to 800nm. The biological performance of the coloured PCBs was assessed using Arabidopsis seedlings (of the standard wild-type ecotype Columbia “Col-0” [17]). Seedlings were grown in 0.2L pots with John Innes No.2 potting mixture and 20% pearlite by volume in a controlled environment chamber (Sanyo Gallenkamp, Loughborough, UK) in 16 h days under cool white fluorescent tubes delivering ~200 umolm^-2^ s^-1^ PAR at the plant canopy. Temperature and RH% set points were 25°C and 80%, respectively. When the plants switched from the vegetative to reproductive phase (identified by a flowering stem emerging from the rosette) coloured PCBs were placed over 10 replicate plants per colour and secured at the bottom of the pot. A standard cellulose PCB and an unbagged control were also included (10 replicates each). The plants were grown in the PCBs until an individual per treatment was identified as having dried siliques and then the PCBs were constricted around the base of the flowering stems with autoclave tape and the unbagged control covered in a glassine bag to prevent seed loss. The rosettes of individuals from all treatments were observed twice weekly until >80% senescence of the rosette was observed, after which watering was withheld for approximately 2 weeks to allow the plant to dry down. Flowering stems were cut off inside the bags, and dried. Seeds from the dry pods were released by gentle manipulation of the bags and seed was separated from the stem and pod material by sieving repeatedly through a 0.8mm sieve onto white paper. Total seed weight per coloured PCB and for the controls was determined on a Mettler Toledo precision balance to 1 mg. Subsamples were taken using doubled sided tape and the adhering seeds were weighed and counted under 10x magnification to calculate the individual seed weights for each treatment.

### Phase II–nonwoven PCB testing physical and biological testing

Materials currently used in the manufacture of commercially available PCBs for Arabidopsis, Miscanthus, wheat and sugar beet were benchmarked for air and water vapour permeability and light transmittance from 350-800nm using a spectral radiometer and integrating sphere (Table 2). These specifications were used in the development of an initial set of three novel nonwoven fabrics, denoted A, B and C1. Fabric A had a thin filtration layer supported by a net like layer which was hot point bonded making the fabric very flexible and easy to tie at the base but requiring internal staking to create space for internal pollen flow. Fabric B was a blend of synthetic and natural wool fibres combined because it was expected wool would improve water vapour exchange. Fabric C1 had the smallest pores, with the lowest air permeability and included viscose to reduce internal humidity accumulation. The specific characteristics of the new nonwoven fabrics in comparison to currently available materials are given in Table 2.

**Table 2:** Details and physical characteristics of the pollination control bags (PCBs) materials developed and evaluated in this study, including standard controls for each of the species to be tested.

ID	Use^¢^	Material type	Thicknesss (mm)	Weight (gm^-2^)	% transmittance*	Air permeability (1m^-2^s^-1^)	Max. pore size (µm)	WVT^$^ (%)
A	Phase II	Polypropylene (PP)	0.40	55.00	38.47	196	44.54	110.44
B	Phase II	Polyester & wool	0.31	97.98	31.39	225	47.05	110.03
C1	Phase II	Polyester & viscose	0.22	92.83	36.13	144	34.45	110.93
C2	Phase III	Polyester & viscose	0.14	80.00	37.29	421	43.52	114.50
D	Phase III	PP	1.04	110.00	20.52	620	296.32	110.49
E	Phase III	Polyester	0.44	110.00	38.78	685	140.08	112.10
Pa	Control	PP (1mm perforated)	0.10	29.00	n/a	363	n/a	n/a
DU	Control	Polyester	0.18	102.58	33.17	78	77.90	99.90
N	Control	Cellulose film	0.06	52.40	86.93	0	0.00	96.29
K	Control	PP non-woven mesh	0.56	103.26	48.67	314	158.60	102.77

^¢^Experimental use in biological testing. The currently used standard pollination control bags for each species were included as controls for Arabidopsis (Pa), Miscanthus (Du), wheat (N) and sugar beet (K) testing.

*Light transmission in the 350-800nm wavelength range

^$^Water vapour transmission

Standard EDANA association (Avenue Herrmann Debroux 46-B-1160 Brussels, Belgium) tests were used to assess physical characteristics of each material. Breathability was assessed using a Textest AG FX3300 mark 4 Labair tester, according to WSP 070.1.R3 (12) at 100 Pa air pressure drop over a 20 cm^2^ test area. Water vapour transmission was tested in accordance with WSP 070.4.R3 which compares water vapour permeability relative to that of a standard polyester monofilament woven fabric which is tested concurrently. Light transmittance of the fabrics was assessed using a Shimadzu UV2600 UV/Vis spectrophotometer with an integrating sphere accessory. A reference beam was applied to the fabric and percentage light transmittance recorded.

All three fabrics were used to create bespoke PCBs for biological testing (Arabidopsis: 2D bags 44cm length x 17cm width; Miscanthus: gusseted 3D bags 75cm length x 15.8cm width; wheat: flat 2D bags 19cm length x 10cm width; sugar beet: 2D bags 139cm length x 60cm width). The currently used standard PCBs were included as controls for each species (Table 2). Total seed weight per plant/cross and germination (expressed as % of germinated seeds after 14 days) were assessed for all species. Additionally, individual seed weight was recorded for Arabidopsis and Miscanthus, total number of seeds for Miscanthus and sugar beet and seed area, width and length for Miscanthus and wheat (assessed using a MARVIN seed analyser (GTA Sensorik GmbH, Germany)). Thousand grain weight (TGW) was also determined for wheat using a MARVIN seed analyser. For sugar beet, assessment of pollen contamination was also made. A full, integrated nutrient, pest and disease management program was used in all experiments to minimize confounding biotic or abiotic stress effects.

For Arabidopsis, the experimental set up was similar to that used in the Phase I colour tests except that when the plants switched from vegetative to reproductive phase the A, B, C1 and control Pa PCBs were placed over eight replicate whole plants and secured at the base of the pots. A plastic stake was used to support all PCBs above the stems and an unbagged control was included (eight replicates). Once the first dried siliques were observed from the unbagged control (17 days after other treatments were bagged), a glassine bag was placed over the flowering stems to prevent seed loss. When a selected individual for each PCB treatment showed the presence of dried siliques, then the PCB was constricted and proceeded to harvest as in Phase I.

The biological testing for Miscanthus used two known sexually cross compatible, but self incompatible, *Miscanthus sinensis* parental clones (Table 1). Field flowering synchronization showed that peak of flowering for Parent A was two weeks earlier than Parent B [18]. Fifty overwintering rhizome clones of Parent A were potted in standard John Innes No. 2 potting compost in late March, 2016. To ensure flowering time alignment between Parent A and B, the start of the growing season was delayed in half the clones (~25 pots) of Parent A by keeping pots of over-wintering rhizomes in a cold room at 4°C until end of June before transfer to the polytunnel where clones of Parent B had been growing since late March. Plants from both clones nearing flowering in Autumn 2016 were transferred from the polytunnel to a naturally lit glasshouse with overnight frost protection heating for crossing. Five replicate controlled crosses were performed by bagging pairs of flowering stems of each clone at the stage when 1–3 cm of panicle had emerged from the shoot’s leaf sheath. PCBs A, B, C1 and the standard PCB (DU) enclosed the synchronized flowering pairs between the 28 September and 10 October 2016. An open pollinated control (unbagged) was also included. Forty days after the flowered stems were bagged (allowing time for cross pollination, seed set and ripening), panicles were cut from the plants and dried for a further 2 weeks to ripen the seed. Spikelets were stripped from the panicles by hand brushing, and then brush threshed through a sieve. The chaff and seed were separated by density. The seed from each treatment was stored at 4°C, before germination tests were performed on wet filter paper at constant 20°C in the dark, and counts based on the appearance of a 1 mm radicle root on days 4, 5, 6, 7, 10, 12 and 14.

Wheat testing was conducted between April and July 2016 and compared 20 replicates of each PCB type A, B and C1 and a standard cellophane control (N) (Table 2). In order to assess temperature effects on pollination given the ears were enclosed at this timepoint, small temperature sensors (Hygrochron iButton DS1923, Maxim Integrated, USA) were included in all biological testing experiments. Two sensors were used per PCB treatment and inserted into PCBs at application and recorded temperature data within the PCB at one minute intervals. A total of nine genotypes (Table 1) were sown in Levingtons Advance M2 potting compost and 2 week old seedlings vernalised at 4°C for eight weeks. Following vernalisation, all seedlings were transferred to 1L pots in a controlled environment glasshouse with a day/night temperature regime of 16 h at 20°C/8 h at 15°C and with supplementary sodium lighting in a randomized complete block design. Crosses were made based on availability and synchronicity of reproductive development and the frequency of use of a genotype as a male or a female were balanced. All plants used as females in crosses were emasculated to remove anthers and the ear covered with a PCB. In order to cross pollinate, bags were removed after 2–3 days and pollen introduced. Bags were then re-sealed at the base of the ear. Seed set was visually assessed after pollination and plants dried down prior to harvest. All pollinated ears were manually harvested and hand threshed.

For sugar beet, vernalized plants of two genotypes (Table 1) were grown for approximately 8 weeks in a greenhouse with supplementary sodium lighting in a randomized block design and five replicate plants per genotype were covered with each PCB along with a standard control (K) before the flowers opened and remained covered until seed harvest 2–3 months later. To determine the potential for external pollen contamination, a row of contrasting red hypocotyl plants with similar flowering times were grown uncovered alongside the PCB covered plants. The presence/absence of the (dominant) red hypocotyls in the progeny was assessed in the germinated seedlings.

### Phase III–increasing non-woven breathability of PCBs

The Phase II biological testing identified a requirement to increase breathability of the PCB fabric by opening up material structure whilst maintaining pollen filtration. A new, thinner version of Fabric C1, denoted C2, and two stiffer, hot point bonded fabrics with up to three times the air permeability of A, B and C1 (Table 2) were developed (denoted D, E). All three fabrics were used to create bespoke PCBs for biological testing of Arabidopsis, wheat and sugar beet and the physical structure of the fabrics were assessed as previously. The Arabidopsis testing was as for Phase II with the exception that the cabinet temperature was lowered from 20°C to 16°C. The wheat testing was conducted as previously with the crossing cycle running from August 2016 to January 2017. The sugar beet testing was as previously but used eight (rather than two) genotypes (Table 1) and inclusion of an unbagged control (five replicates).

### Statistical analysis

For the biological testing Phase II and III experiments all trait data was analysed using Genstat (16^th^ Edition; VSN International, Hemel Hempstead, UK). As the Phase II and III experiments were separated in time, data was analysed separately for each phase. For Arabidopsis and wheat a one-way ANOVA was used to detect statistical differences due to PCB treatment. For Miscanthus and sugar beet a two-way ANOVA was used to detect statistical significance due to PCB treatment and genotype. The distribution of residuals was visually inspected for all trait/species combinations. Where residuals were not normally distributed data was transformed using log(10)-transformation and back-transformed means are reported. Regression analysis was used to determine trait-by-trait and trait-by-physical characteristic (Table 2) correlations. Results were plotted in R (R Core Team, 2015).

### Environmental decoupling experiments

The EDANA standard fabric tests were supplemented with further whole bag tests to compare the water vapour transmission rate and the interactions between light transmission and temperature of the novel PCBs and control materials used in Phase II and III. The tests used PCBs with internal dimensions 15 x 40 cm with twelve calibrated temperature and humidity probes (CS 215 logged every 10 secs by a CR800, Campbell Scientific, Loughborough, UK) enclosed in PCBs supported on a retort scaffold. To determine water vapour transmission, a steady baseline at ambient laboratory humidity (time zero) was recorded for 5 minutes after which water vapour mist (with particles around 3–4 um) produced by an ultrasonic water vapour generator (Piezoelectric MDK6-24C, MEGIC, Guangdong, China) was fed evenly over baffles into a temperature unregulated tent containing the bagged CS 215 probes. The humidifier increased the relative humidity rapidly, from approximately 40% to 90% in 3 minutes, and >90% in 10 minutes. PCBs could be transferred on and off each probe allowing for replicated runs with re-randomised positions to reduce experimental error. The time in minutes for a change of vapour pressure deficit from 12 to 2 (hPa) was recorded for each PCB type over seven replicate runs, with one random control probe and one fixed central control probe without a PCB. An additional glassine (G) control PCB was included in the experiments.

To determine the interaction between light transmission and temperature, the probes were moved to a controlled environment chamber (Sanyo Gallenkamp, Loughborough, UK) with constant temperature (20°C) and relative humidity (40%). The PAR at the top of the bags (provided by high intensity white florescent lamps and supplementary tungsten bulbs) was 788 umol m^-2^ s^-1^. Maximum temperature changes within the PCBs were recorded after 1 h under the lights having stabilised for 1 h without lighting. The ambient temperature in the cabinet was monitored at each position the difference from the mean cabinet temperature was added to the temperatures of all the sensors to account for any spatial temperature variation. The experiment was run 11 times randomising the position of each PCB and two controls in the cabinet each run. The data from both experiments were analysed in R [19]. Normality was assessed and data transformed if required and then analysed using a one-way ANOVA (residuals checked with a Shapiro-Wilk test of normality), followed by a Tukey’s HSD (honest significant difference) test.

## Results and discussion

### Pollen characterization

**Fig 1:**
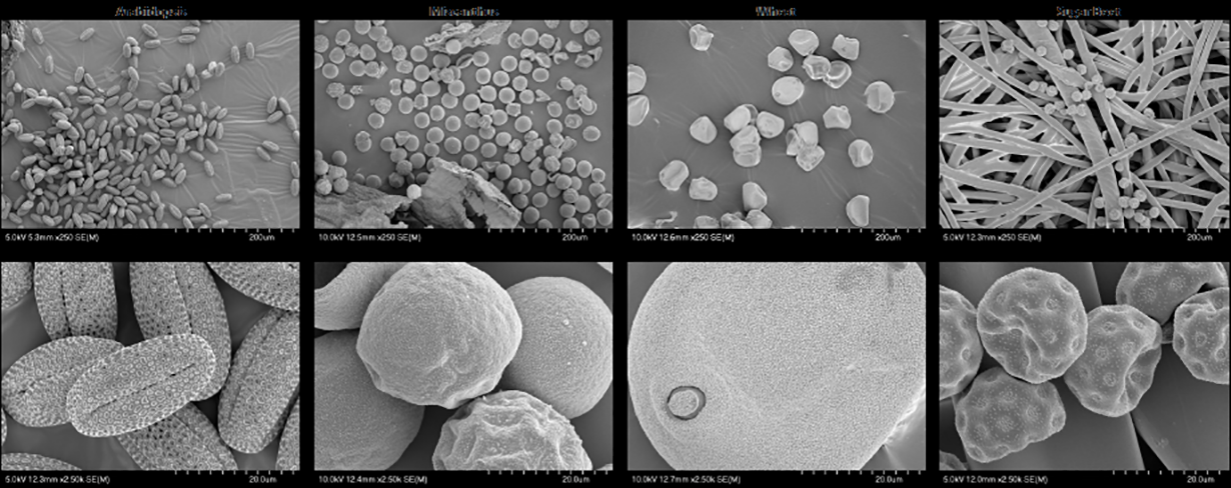
**Scanning electron microscope images of pollen from Arabidopsis (*Arabidopsis thaliana*), Miscanthus (*Miscanthus sinensis*), wheat (*Triticum aestivum*) and sugar beet (*Beta vulagaris*) at x250 (top panel) and x 2500 (bottom panel)**. The sugar beet pollen was imaged over PCB B.

Pollen SEM images were produced at x250 and x2500 magnification (Fig 1). Three species had spherical pollen while Arabidopsis pollen was prolate spheroid shaped. Pollen size was determined (Table 1) and used to inform the design of the nonwoven fabrics tested in Phase II.

### Phase I PCB colour testing

**Fig 2:**
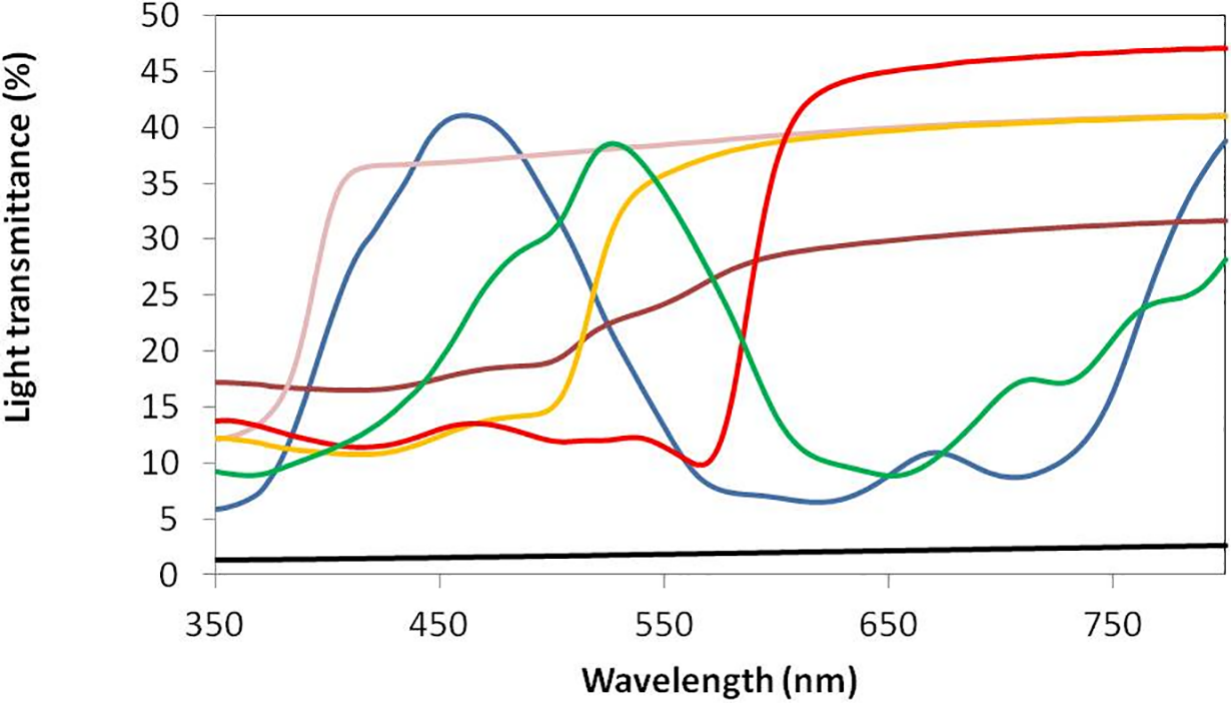
Light transmission (%) measured for coloured polypropylene fabric in 5nm steps from 350 to 800nm.

**Fig 3:**
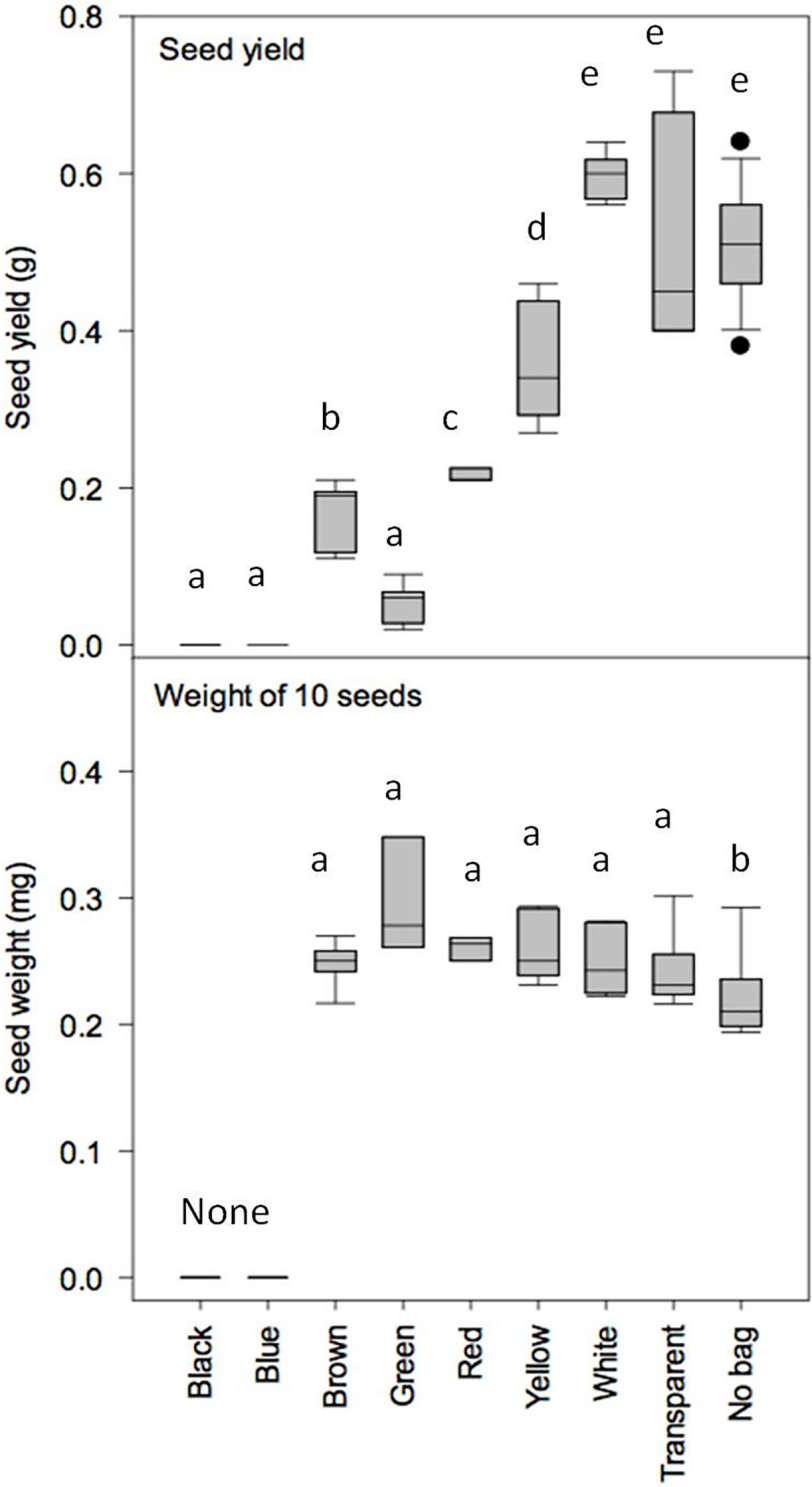
**Seed yield of Arabidopsis in different coloured polypropylene PCBs showing total seed yield (g) per plant (top panel) and the average weight of 10 seeds (bottom panel)**. Different lower case letters denote significant differences between treatments.

The light transmittance (%) across the 350 to 800nm wavelength range showed different colour PCBs giving strongly contrasting wavelength filtering (Fig 2). Black screened out all wavelengths and blue had high transmission in the range 400-500nm, and then become opaque to red light. Conversely, red PCBs had low transmission until 550nm, after which the transmission was the highest of all colours tested. Brown PCBs had generally low transmission with little differential filtering of wavelengths. White bags filtered wavelengths below 400nm, but all wavelengths were equally transmitted above 400nm. When biologically tested using Arabidopsis, no seeds were obtained from the black or blue PCBs (Fig 3). Green PCBs produced low yields of heavy seeds. Red and yellow PCBs had intermediate yield and seed weights. There was no significant difference in seed yield for the white PCB or standard cellulose control (Fig 3). In general, coloured PCBs were detrimental to seed yield and colours with non-selective wavelength filtering performed best. Consequently, white nonwoven fabrics were used for subsequent material development in Phase II and III.

### Physical PCB testing

**Fig 4:**
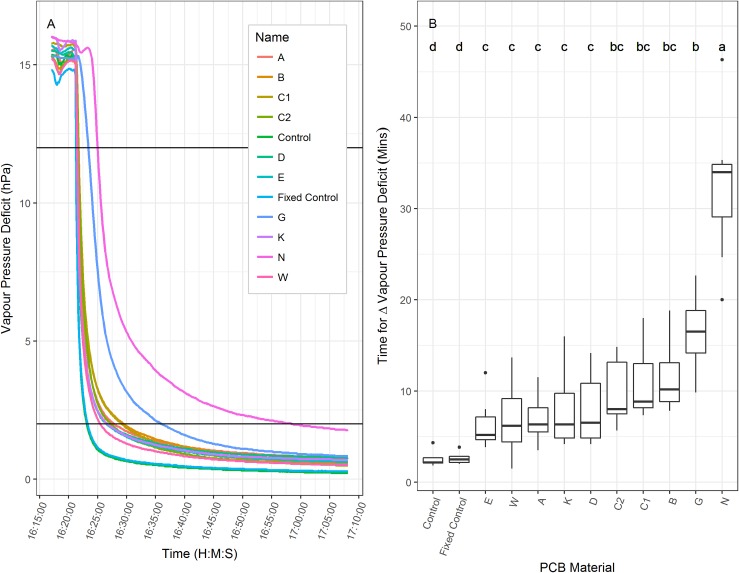
Internal vapour pressure deficit (hPa) response rates to rapid changes in humidity outside the PCBs. The left panel shows the time course of one 30 minute transition from ambient laboratory humidity (dry) to air wetted rapidly by ultrasonic humidification. The right panel is a box plot of the average time for the transition from 12 to 2 hPa for each bag type (of 7 replicate transitions). Significant differences are denoted by different lower case letters.

Material benchmarking revealed a range of physical variation between the novel and control materials (Table 2). A number of the physical characteristics were significantly correlated (Table A in S1 File), with the strongest (positive) correlation recorded between material thickness and pore size (P<0.001). The weights of the materials ranged from 29gm^-2^ (Pa) to 110gm^-2^ (D, E) and were significantly correlated with light transmission (negative), air permeability (positive) and pore size (positive). Percentage light transmission ranged from 20.52% (D) to 86.93% (N). With the exception of D, all other novel materials had light transmissions in the 30% range which were generally lower than the control materials (with the exception of DU). Air permeability ranged broadly from 0 (N) to 685m^-2^s^-1^ (E) and was correlated with both thickness and weight. The Phase III materials had higher permeability (range 421 to 685 m^-2^s^-1^) than the Phase II materials (range 144–225 m^-2^s^-1^). The water vapour transmission of all novel materials was higher than all of the control materials (Table 2). A rapid change in external humidity caused changes in internal PCB humidity (vapour pressure deficit (VPD) hPa; Fig 4) under laboratory conditions. Transition rates (in minutes) were also calculated using the thresholds 12 (dry) to 2 (wet) hPa (Fig 4). All bagged temperature and humidity probes responded more slowly (>5 minutes) to the rapid increase in external humidity compared to the unbagged controls (<3 minutes).

**Fig 5:**
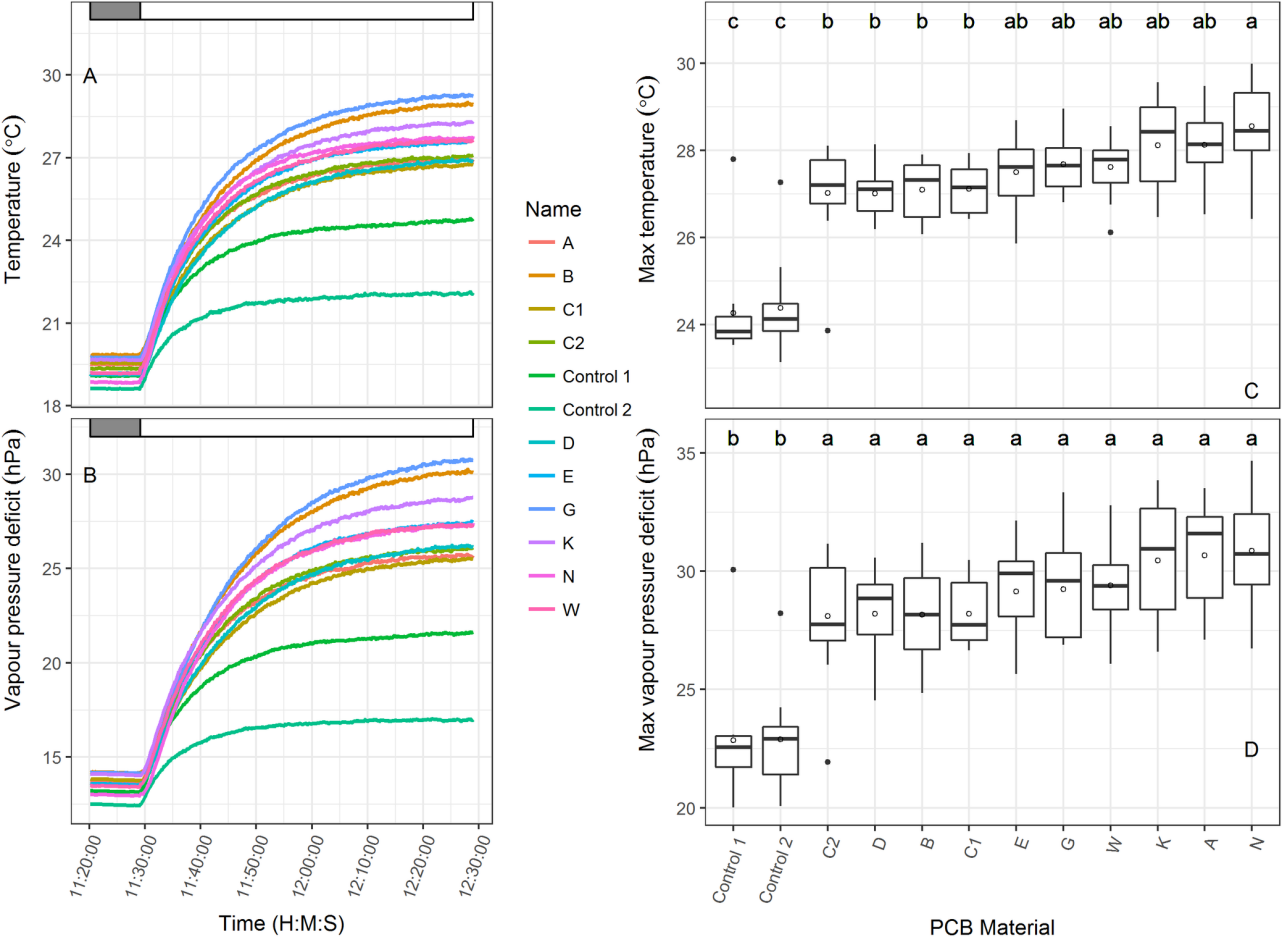
Internal temperature (a) and vapour pressure deficit (hPa, b) response rates to a transition from dark to light (0 to 880 umolm^-2^s^-1^) above the pollination control bags. Box plots of the average maximum temperature (c) and VPD (d) are shown for each bag type. Significant differences are denoted by different lower case letters for temperature and VPD and are based on 11 replicates.

Significant variation was also detected in internal PCB temperature and VPD measured over a time course in controlled environment conditions (Fig 5). Following a dark period the lights were switched on (indicated by dark to light shading in Fig 5) and temperature increased rapidly for 10 minutes, and gradually for another 20 minutes (Fig 5). The unbagged control probes stabilized at about 22°C after 30 minutes, while those in PCBs ranged from 38 to 31°C after an hour. The PCBs B, C1, C2, and D had significantly lower temperature increase than N, while the other PCBs were only significantly different from the controls. The higher temperatures in PCBs were found to significantly increase the VPD from ~23 hPa for the controls to ~29 hPa (Fig 5).

### Biological PCB testing

#### Arabidopsis

The amount of seed produced (total and individual seed weight) and germination in all treatments was lower in Phase III compared to Phase II presumably a result of the reduced temperature (Phase II 20°C; Phase III 16°C) (Table 3). However, despite the magnitude differences, across the Phase II and III Arabidopsis testing significant differences were detected for total and individual seed weight (Table 3) but there was no significant variation for germination. A significant positive correlation was detected between individual seed weight and germination in both Phase II (P = 0.045) and III (P = 0.008) (Table B in S1 File). In Phase II, the control PCB (Pa) had very low total seed weight but the highest individual seed weight. PCB A had the highest total seed weight but intermediate individual seed weight. PCBs B, C and the unbagged control had intermediate total and individual seed weights. Germination did not vary significantly between the treatments. In Phase III, the unbagged control had the highest total seed weight and correspondingly high individual seed weights. The lowest total seed weight was detected for PCB E, although it gave intermediate individual seed weight. Reflecting the discrepancy in the relationship between individual and total seed weight, there was a significant (P<0.001) difference in Phase II, but not in Phase III. In order to assess the relationship between the biological and physical characteristics, correlation P-values were calculated for PCB A, B, C1, C2, D and E in combination (Table 4). The control PCB Pa and the unbagged control were excluded as no physical characteristics were quantified. Significant negative correlations with air permeability were detected for total seed weight (P = 0.005), individual seed weight (P = 0.048) and germination (P = 0.017) with lower permeability giving higher total and individual weights, and as a result, higher germination.

**Table 3:** Arabidopsis biological testing results as means of traits and significance values (with least significant differences) in Phase II and Phase III testing with significant results highlighted in bold.

PCB	Phase	Total seed weight (g)	Individual seed weight (g)	Germination %
A	II	1.096	0.0224	93.13
B	II	0.890	0.0221	97.62
C1	II	0.943	0.0243	92.88
Pa	II	0.162	0.0334	91.38
No bag	II	0.894	0.0206	96.62
*P-value* *(l*.*s*.*d*.*)*		***<0*.*001 (0*.*1272)***	***<0*.*001*** ***(0*.*0021)***	*0*.*062* *(4*.*857)*
C2	III	0.400	0.0198	72.96
D	III	0.363	0.0181	73.16
E	III	0.247	0.0207	73.62
Pa	III	0.344	0.0219	73.79
No bag	III	0.435	0.0218	74.46
*P-value* *(l*.*s*.*d*.*)*		***0*.*016*** ***(0*.*1087)***	***0*.*004*** ***(0*.*0021)***	*0*.*119* *(1*.*198)*

**Table 4:** Correlation values (R^2^) and corresponding P-values (in parentheses) between biological performance and physical PCB characteristics for Arabidopsis Phase II and III testing, with significant values highlighted in bold.

	Biological property		
Physical property	Total seed weight (g)	Individual seed weight (mg)	Germination (%)
Thicknesss (mm)	0.132 (0.479)	0.427 (0.159)	0.148 (0.451)
Weight (gm^-2^)	0.412 (0.170)	0.137 (0.470)	0.164 (0.426)
% transmittance	0.061 (0.637)	0.339 (0.225)	0.041 (0.699)
Air permeability (1m^-2^s^-1^)	**0.883 (0.005)**	**0.665 (0.048)**	**0.793 (0.017)**
Max. pore size (um)	0.381 (0.192)	0.594 (0.073)	0.374 (0.197)
Water vapour transmission (%)	0.316 (0.246)	0.099 (0.544)	0.412 (0.169)

#### Miscanthus

A two-way ANOVA detected no significant interaction between genotype and PCB type (Table C in S1 File). A significant difference was detected between genotypes for seed width (P = 0.017) with genotype Parent A having wider seed compared to Parent B for all PCB treatments except PCB C. The highest seed set was found in the unbagged open pollinated plants. No significant correlations were detected for any of the physical and biological properties of crosses made using any of the PCBs.

#### Wheat

**Fig 6:**
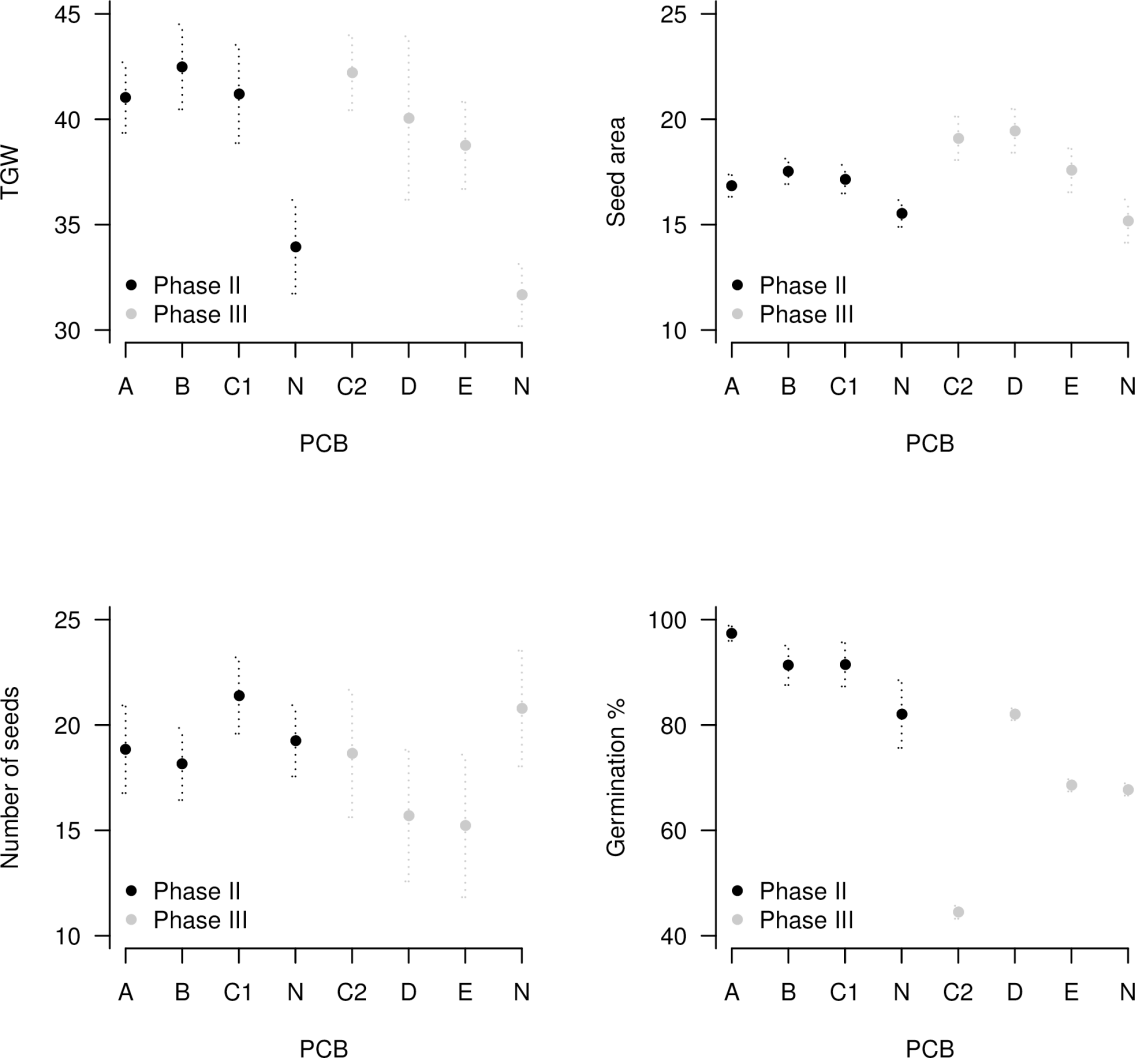
Wheat biological testing results from Phase II and III showing significant differences between thousand grain weight (TGW) in both phases and seed area in Phase III.

Over both phases of biological testing, 158 test crosses were assessed. Statistical analysis by PCB type showed that there were significant differences for TGW in both phases (Phase II P = 0.020; Phase III P = 0.023) with the control bag (N) producing lower TGWs compared to all of the novel PCBs (Table D in S1 File). In Phase II no other significant differences in biological performance were observed. In Phase III, additional significant variation was revealed for seed area (mm), width (mm) and length (mm) (all log10 transformed; P<0.001, 0.002, <0.001, respectively; Table D in S1 File). All of the significant differences supported the superior performance of the novel PCBs in comparison to the control PCB (N). Although there were no statistical differences in biological performance of the novel PCBs, additional variation was observed, with results for number of seeds, seed area, TGW and germination percentage displayed in Fig 6. Significant correlations were detected between the physical PCB properties and biological performance (Table 5). TGW, which was significant in both testing phases, was significantly negatively correlated with % light transmittance (P = 0.001; R^2^ = -0.84) and positively with water vapour transmission (P<0.00; R^2^ = 0.86). As this was proposed to be a temperature effect, internal PCB temperatures were compared over the experimental timeframes, showing that control N (cellophane) had the highest recorded enclosed ear temperatures and greatest internal temperature variance in both phases (Table E in S1 File). The rate of contamination by ambient pollen was established in Phase III by comparing emasculated ears covered by PCBs C2, D, E and N with an unbagged (emasculated) ear. Seven seeds developed in the uncovered ear but no seeds developed in any of the other treatments and it was therefore concluded that all PCBs effectively filtered external pollen.

**Table 5:** Wheat correlation R^2^ values (with corresponding significance P-values) for physical and biological characteristics of novel and control PCBs in Phase II and III. Significant values are highlighted in bold.

Physical characteristic	Total seed weight (g)	TGW	Seed area (mm)	Width (mm)	Length (mm)	Germination %
Thickness (mm)	0.102 (0.440)	0.136 (0.370)	0.445 (0.071)	0.129 (0.382)	**0.568 (0.031)**	0.060 (0.559)
Weight (gm^-2^)	0.117 (0.408)	0.336 (0.132)	**0.537** (**0.039**)	0.276 (0.181)	**0.504 (0.049)**	0.001 (0.959)
% transmittance	0.066 (0.538)	**0.835 (0.001)**	**0.747 (0.006)**	**0.688 (0.011)**	**0.584 (0.027)**	0.024 (0.717)
Air permeability (1m^-2^s^-1^)	0.342 (0.128)	0.229 (0.230)	**0.649 (0.016)**	0.261 (0.195)	**0.773 (0.004)**	0.092 (0.464)
Max. pore size (µm)	0.237 (0.221)	0.083 (0.489)	**0.506 (0.048)**	0.113 (0.416)	**0.682 (0.012)**	0.001 (0.956)
WVT (%)	0.103 (0.439)	**0.864 (< .001)**	**0.695 (0.010)**	**0.732 (0.007)**	**0.513 (0.046)**	0.005 (0.870)

#### Sugar beet

Two different sets of germplasm were used in the Phase II and Phase III self pollination testing, and because of this both analyses accounted for genotype and PCB treatment. The treatment and genotype means for the two contrasting genotypes (G_01, G_98), and significance values for Phase II are given in Table 6. No significant genotype x PCB interactions were detected but genotype and PCB treatment both significantly impacted biological performance for all assessed traits (total seed weight, number of multigerm seeds, germination and number of seedlings). Genotype G_01 consistently outperformed G_98 for all assessed traits. For total seed weight the control PCB (K) gave superior biological performance compared to all other bags for G_98. For G_01 PCB K and A had similar performance, and both were superior to B and C1. The control (K) significantly outperformed the novel PCBs for total number of seeds for G_98 but was not significantly different to PCBs A and C1 with all three performing significantly better than PCB B. For germination, although PCB B had the lowest performance for G_01 it wasn’t significantly different from any of the other PCBs. For G_98 germination was generally low with the control (K) giving the highest germination (56%) compared to C1 and B which both outperformed A. Number of seedlings (derived as number of seeds x germination rate) is an important characteristic in breeding programmes and can detect performance differences. For example, for G_01 the control K had the highest number of seedlings, although was not significantly different to C1. This was in contract to the results for G_98 where control PCB K outperformed the novel materials by almost two-fold.

**Table 6:** Sugar beet biological testing results for Phase II incorporating genotype and PCB treatment effects. Two contrasting genotypes were used in this experiment (G_01, G_98) and no significant genotype x PCB treatment effects detected.

	Total seed weight (g)		Number of seeds		Germination		Number of seedlings	
PCB	G_01	G_98	G_01	G_98	G_01	G_98	G_01	G_98
A	7.15	4.386	855.8	431.1	74.62	33.42	618.20	160.50
B	4.74	3.1	647.3	334.1	73.21	38.37	493.90	143.70
C1	6.30	3.533	825.5	363.5	79.11	41.57	684.60	157.10
K	7.37	5.746	894.6	548.1	77.36	55.59	706.00	303.40
***P-value*** ***l*.*s*.*d***	***<0*.*001*** ***(1*.*74)***		***<0*.*001*** ***(194*.*10)***		***<0*.*001*** ***(12*.*25)***		***<0*.*001*** ***(170*.*1)***	

In Phase III eight contrasting genotypes (denoted 1–8) and an unbagged control were used. Both total seed weight and germination had non-normal distribution of residuals so log(10)-transformations were used for analyses (presented in Table F in S1 File). Significant interactions between genotype and PCB treatment were detected for number of multigerm seeds and germination (P<0.001 in both cases) whilst for total seed weight and number of seedlings both genotype and PCB treatment were significant (P<0.001 in both cases). Much of the variation arose from the unbagged control which gave large variation across the contrasting genotypes but was statistically superior for total seed weight and number of seeds with lower germination and generally low number of seedlings (Table F in S1 File). With the unbagged treatment removed the statistical analysis identified the same significant interactions. Ranking of Phase III unbagged performance (Table G in S1 File) for each genotype revealed interesting variable PCB effects. Among the five genotypes with the highest unbagged seedling potential, four (1, 3, 6, 7) showed PCB K to have the best across-trait performance. For one genotype (4) PCB E had consistently highest performance and this genotype had unbagged seedling potential below the above four genotypes. The remaining three genotypes (2, 5, 8) had the lowest unbagged seedling potential and showed variable PCB performance across traits, although PCB E performed consistently well for number of seedlings. Overall PCB D had the weakest biological performance and C1 was inconsistent, although it produced high total seed weights for genotype 2 and 5, along with high seed numbers for genotypes 5 and 8.

In both phases, total seed weight was correlated with number of multigerm seeds (P<0.001). In Phase III germination rate was also significantly correlated with total seed weight (P = 0.002) and number of seeds (P<0.001) but this wasn’t observed in Phase II. When physical and biological properties were correlated, a significant positive correlation between % light transmittance and total seed weight and number of seeds (both P = 0.015) was detected in Phase II for G_98, but not for G_01 (Table H in S1 File). Several genotype-specific correlations were also observed in Phase III for all biological and physical characters with the exception of water vapour transmission (Table I in S1 File). Notably, the seed and seedling performance of genotype 7 was significantly influenced by light transmission. Pollen contamination, assessed using the red hypocotyl phenotype, was less than 0.5% from all PCBs tested and a high rate of contamination (14%) was recorded for the unbagged plants.

## Discussion

This is the first study to systematically manufacturer and test bespoke PCBs. We hypothesized that a novel breathable ‘nonwoven’ fabric optimal for both pollination and seed set in multiple plant species could be developed. This would have an industrial advantage as a single fabric for PCB production could be rapidly upscaled. We tested a range of novel nonwoven fabrics using a range of fibre-type, layering and bonding methods. Through iterative material development coupled with physical and biological testing we determined that nonwoven fabrics outperform paper and cellophane, but that the choice of optimal PCB is crop, genotype and/or environment specific. A different nonwoven fabric gave superior performance for three of the four species tested in this study, and we propose this to be the result of crop-specific pollination mechanisms and variation in the environmental conditions used for crossing and/or seed production.

Initial colour testing demonstrated that light passing through PCB material affects seed yield. Black and blue PCBs had low light transmittance beyond 550nm and plants produced no seeds. Green, red and yellow produced small but heavy seeds, possibly as a stress response [20–22]. Although seed weight is a yield component it cannot always be singularly applied to assess performance, as high individual seed weight was associated with low yield in experimental conditions. The best performance in seed yield was recorded for the unbagged, transparent and white PCBs. As it is not possible to produce transparent nonwoven material (at a realistic price point), white was chosen as the most suitable colour for novel PCB development and is recommended for future studies on the influence of materials on pollination.

Overall the six novel PCBs created in this study outperformed the standard control bags (with the exception of genotype-specific results in sugar beet). This is consistent with previous attempts to generate or identify existing materials with superior pollination performance. McAdam et al. [23] demonstrated the potential of non-woven fabrics over paper bags in the grass *Lolium perenne* breeding. More recently, Adhikari et al. [24] showed that polyester PCBs were more reliable in reducing outcrossing and physical contamination in switchgrass (*Panicum virgatum*) breeding. Polyester micromesh has also been demonstrated to improve pollination control in switchgrass [25] and Tyvek^®^ housewrap [26], a spunbonded, high-density polyethylene fabric developed as a material for air infiltration in house construction was tested in sorghum showing a trade-off between overheating and pollen exclusion [12]. Unlike previous studies, our study is the first to iteratively create and biologically test novel non-woven fabrics for improving controlled cross pollination across multiple species. The novel fabrics varied for a number of physical characteristics (summarized in Table 2) compared to existing materials and all had superior water vapour transmission rates. Across the biological testing phases no single “winner” was identified, disproving the hypothesis that a single novel nonwoven fabric could be identified. However, interesting results were recorded across the biological testing phases.

In Arabidopsis, air permeability had a significant influence on total seed weight, individual seed weight and germination. Lower permeability resulted in higher total seed weight indicating that high rates of air flow within the PCB reduced self pollination efficiency. Previous work has shown that pollen tube growth in Arabidopsis initiates within 5° to the closest point of contact with the stigma papilla and that this becomes disrupted in the presence of high (90%) humidity reducing pollination efficiency [27]. Since air flow rates were high in the controlled environment cabinet used, we propose that air flow through the PCB caused similar disruption. In self-pollinated Arabidopsis, PCBs are generally used to prevent contamination of research facilities with seed that is easily released from the drying siliques. As a high proportion of Arabidopsis research utilizes transgenic lines, in the UK it is a requirement for compliance that the seed and pollen be contained. Most publications do not report on the type of materials used to enforce containment, although the Arabidopsis Biological Resources Stock Centre recommends the use of polypropylene plastic bags or plastic floral sleeves (https://abrc.osu.edu/seed-handling). In two out of the three rounds of materials testing in this study cellophane (plastic) PCBs produced the lowest total seed yields but the largest seeds and novel PCBs had a more consistent impact on fecundity. An inverse relationship has been previously reported between seed number and seed weight in Arabidopsis [28] with higher numbers of seeds within a developing silique negatively impacting size [29].

In Miscanthus and sugar beet, the experimental findings were confounded by genotypic differences. No significant differences in seed production were found between Phase II novel PCBs A, B and C1 in Miscanthus but all values were lower than the unbagged open pollinated controls. In Miscanthus, flowering synchronisation between the two parents is difficult to control. Lower seed set in pairwise crosses in PCBs result from less wind, less pollen, and higher temperatures. To date agricultural production of Miscanthus has been largely based on the vegetative propagation of the triploid interspecies hybrid (*M*. *x giganteus*). However, rapidly multiplied seeded hybrids are needed to support the widespread uptake and expansion of Miscanthus as a bioenergy feedstock [30]. The continued success of the breeding of improved seeded hybrids relies, in part, on the efficiency of controlled cross-pollinations using PCBs, which provide seed for progeny tests needed to identify potential combinations producing outstanding seeded hybrids. Therefore, continued efforts are required in the re-design of PCB materials supporting pollination efficiency.

In the sugar beet self-pollination experiments, the standard PCB K, an existing proprietary non-woven, outperformed the Phase II PCBs (A, B, C1; except for G_01 germination). A similar level of performance was observed in Phase III but was confounded by the genotype x PCB interaction and there was evidence for improved performance of novel material E. A positive relationship between light transmittance was observed for the lower performing genotype (G_98) in Phase II, with low light transmittance reducing total seed weight and number of seeds. In Phase III control K gave superior biological performance for high potential genotypes (determined as those with the highest unbagged characteristics). However, novel PCB E outperformed K for the moderate potential genotype 4 and there was evidence for improved seedling number in the low performance genotypes. Therefore, although the current proprietary standard K ensures suitable seed outputs there is scope to further refine materials to support the performance of lower potential genotypes. Controlled self-fertilisation is an important component of sugar beet breeding and an early control recommendation stipulated that a space equivalent to a US city block should separate plantings [31]. Brewbaker [31] field tested a range of materials, including heavy cotton cloth, grocery bags, vegetable-parchment and cellophane reporting genotypic differences due to the fitness of the selfed individuals. As the system for sugar beet pollination control requires that the PCB covers the entire plant, the pursuit of superior PCB fabrics for optimising seed performance will continue. Importantly, our results show that there is particular potential for novel non-woven materials (particularly fabric E) to improve the viable seed outputs from intermediate or low performing genotypes. This is an important output for breeding as not all genotypes have equal performance in terms of seed traits, but may offer advantageous agronomic characters. Therefore these results show the potential to boost seed and seedling production across a broader range of genotypic performance.

Of the four species in this study, the pollen from sugar beet is the smallest (~20–25 μm). Tests included an externally placed naturally occurring red hypocotyl control to detect contamination. This showed that PCBs of non-woven fabrics with maximum pore sizes greater than the pollen diameters produced no contaminated seedlings. We propose that the lack of contamination detected is the result of the complexity of the material, with a physically tortuous path through the fibrous mesh ensuring that external pollen cannot easily pass through the fabric. This ‘effective filtering’ may not be sufficient for assured containment purposes where an impermeable material is required, although the trade-off is clearly in pollination performance. Here we have identified fabrics that provide a level of filtering that co-optimises exclusion of foreign pollen and pollination performance in a breeding context. This may not translate to the field, as previous work has recommended that maximum pore sizes be kept just under the pollen size of the crop [25].

The key finding in the wheat biological testing was the impact of PCB type on TGW, proposed to be a response to changes in the micro-environment around the developing seed. Bentley et al. [32] reported that TGW had low heritability in field trials of European winter wheat grown across a wide range of agro-environments. Here the PCBs were only placed over the ears for a limited time period, therefore having a relatively short timeframe to influence seed production traits. Therefore, the differences recorded give an interesting new insight into TGW (in controlled conditions) as they are in contrast to previous reports that they are fixed during the growth cycle [33]. In Phase III specific seed traits including area, width and length were also significantly increased by the novel PCBs indicating that these traits exhibit environmentally driven plasticity. Light transmittance negatively impacted TGW, with low light giving higher TGW, whilst water vapour transmission had a positive impact. This suggests that low light and higher humidities increase TGW, presumably by reducing temperature (as discussed above). This information could be used to extend the current product development. In the wheat testing, the novel PCBs significantly outperformed the standard cellulose film bags demonstrating a clear opportunity to improve wheat pollination and seed set through the adoption of nonwoven PCBs.

The differences recorded between PCB-type and species under test were impacted by environmental interactions. Correlations between physical and biological traits give some preliminary indication that different properties have greater impacts in different species (air permeability in Arabidopsis; light transmission and water vapour transmission in wheat). In order to understand the baseline properties of novel and control materials, a range of physical tests were developed and deployed. Transitions from low to high humidity show that nonwovens are more highly permeable to water vapour when compared to traditional PCB materials such as cellophane. This confirms the value of non-woven PCBs. In general, the more light transmittance through the PCB, the greater the heating during bright parts of the day. The climate chamber equipment to measure temperature increases from dark to light showed heating effects in all novel PCBs were indistinguishable, but heated on average 36% less than cellophane. In the wheat experiments, low light transmittance was correlated with high TGW. Pollination in general is known to be reduced by high temperatures [34–35] with wheat being specifically sensitive to high temperature at pollination [36]. Therefore, a key design factor for future wheat PCBs, particularly those to be used in glasshouses where temperatures rise rapidly during sunny days, should incorporate this physical property. Further work is required to understand field performance of the novel materials as more complex weather and environmental variables are likely to impact on performance.

This study has, for the first time, iteratively developed, manufactured and tested a range of novel nonwoven fabrics as bespoke PCB materials for crop breeding. No single nonwoven material had superior performance across the diverse species tested, indicating that further optimization of PCB materials should be species-specific, rather than species-generic. However, in almost all cases the novel nonwovens outperformed standard controls, indicating that there is significant scope to improve pollination and seed set through material improvement. As the complexities of the biological and physical factors underlying seed set and viabilities were observed to be large, future studies should focus further on optimizing the performance of individual crop species. Such future work could prioritise crops with a large demand for PCBs (enabling industrial scaling) and small pollen (requiring effective filtration), such as sugar beet. This could be combined with further studies on the viability and longevity of pollen within PCBs, their design and use, further elucidating the potential gains from PCB innovations for breeding.

## Supporting information

S1 File Supporting information: **Table A.** Results of physical characteristic testing with P-values in bold denoting significant correlations between physical properties of novel non-woven and control materials (described in Table 2) used in Phase II and III. **Table B**. Arabidopsis trait-by-trait correlations for Phase II and Phase III. Significant correlations are highlighted in bold. **Table C.** Miscanthus biological testing results (Phase II only) with significant P-values highlighted in bold **(**along with average least significant difference given in parentheses). **Table D.** Wheat biological testing results as means of traits and significance values in Phase II and Phase III testing with significant results highlighted in bold **(**along with average least significant difference given in parentheses).**Table E.** Sugar beet biological testing results as means of traits and significance values in Phase III testing **(**along with average least significant difference given in parentheses). **Table F.** Internal PCB temperatures recorded using iButton sensors enclosed in test PCBs over both experimental testing phases (Phase II: 4096 observations; Phase III: 4077 observations). **Table G.** Unbagged performance of Phase III sugar beet genotypes for all biological traits. **Table H.** Correlation between physical and biological properties for sugar beet genotypes G_01 and G_98 Phase II testing with significant results highlighted in bold. R^2^ correlation values are given in parentheses for significant results. **Table I.** Correlation between physical and biological properties by genotype (denoted 1–8) for sugar beet Phase III testing with significant results highlighted in bold. R^2^ correlation values are given in parentheses for significant results.
(DOCX)
