# Supplementary material for: Investigating the potential of novel non-woven fabrics for efficient pollination control in plant breeding
Source: PLoS One. 2018 Sep 28;13(9):e0204728. doi: 10.1371/journal.pone.0204728 (PMC6161889; doi:10.1371/journal.pone.0204728)
Supplement: S1 File — Supporting information: Table A. Results of physical characteristic testing with P-values in bold denoting significant correlations between physical properties of novel non-woven and control materials (described in Table 2) used in Phase II and III. Table B. Arabidopsis trait-by-trait correlations for Phase II and Phase III. Significant correlations are highlighted in bold. Table C. Miscanthus biological testing results (Phase II only) with significant P-values highlighted in bold (along with average least significant difference given in parentheses). Table D. Wheat biological testing results as means of traits and significance values in Phase II and Phase III testing with significant results highlighted in bold (along with average least significant difference given in parentheses).Table E. Sugar beet biological testing results as means of traits and significance values in Phase III testing (along with average least significant difference given in parentheses). Table F. Internal PCB temperatures recorded using iButton sensors enclosed in test PCBs over both experimental testing phases (Phase II: 4096 observations; Phase III: 4077 observations). Table G. Unbagged performance of Phase III sugar beet genotypes for all biological traits. Table H. Correlation between physical and biological properties for sugar beet genotypes G_01 and G_98 Phase II testing with significant results highlighted in bold. R2 correlation values are given in parentheses for significant results. Table I. Correlation between physical and biological properties by genotype (denoted 1–8) for sugar beet Phase III testing with significant results highlighted in bold. R2 correlation values are given in parentheses for significant results. (DOCX) [file pone.0204728.s001.docx]

**S1 File. Supporting information:**

**Table A.** Results of physical characteristic testing with P-values in **bold** denoting significant correlations between physical properties of novel non-woven and control materials (described in Table 2) used in Phase II and III.

| Physical property | Thickness | Wt | Transmittance | Perm | Pore_size | WVT |
| --- | --- | --- | --- | --- | --- | --- |
| Thickness |  |  |  |  |  |  |
| Wt | 0.083 |  |  |  |  |  |
| Transmittance | 0.098 | **0.036** |  |  |  |  |
| Perm | **0.021** | **0.046** | 0.104 |  |  |  |
| Pore_size | **<.001** | **0.022** | 0.111 | **0.010** |  |  |
| WVT | 0.233 | 0.189 | 0.113 | **0.046** | 0.292 |  |

**Table B**. Arabidopsis trait-by-trait correlations for Phase II and Phase III. Significant correlations are highlighted in **bold**.

|  | Total seed weight (g) | | Individual seed weight (mg) | |
| --- | --- | --- | --- | --- |
|  | Phase II | Phase III | Phase II | Phase III |
| Individual seed weight (mg) | **<.001** | 0.838 |  |  |
| Germination % | 0.319 | 0.838 | **0.045** | **0.008** |

**Table C.** Miscanthus biological testing results (Phase II only) with significant results highlighted in **bold (**along with average least significant difference given in parentheses).

|  | Seed number | | Average seed number (mg) | | Mean seed weight (mg) | | Mean seed area | | Mean seed width | | Mean seed length | |
| --- | --- | --- | --- | --- | --- | --- | --- | --- | --- | --- | --- | --- |
| PCB | A | B | A | B | A | B | A | B | A | B | A | B |
| A | 8.80 | 11.25 | 7.64 | 8.45 | 0.81 | 0.63 | 1.62 | 1.43 | 0.84 | 0.75 | 2.20 | 2.10 |
| B | 1.50 | 16.00 | 1.30 | 10.58 | 0.88 | 0.64 | 1.70 | 1.42 | 0.83 | 0.78 | 2.33 | 2.12 |
| C1 | 13.50 | 7.00 | 17.90 | 4.78 | 0.69 | 0.77 | 1.45 | 1.52 | 0.80 | 0.80 | 2.15 | 2.18 |
| W | 6.33 | 13.33 | 5.67 | 9.30 | 0.84 | 0.66 | 1.57 | 1.43 | 0.83 | 0.77 | 2.17 | 2.17 |
| Unbagged | 6.00 | 10.60 | 3.00 | 5.90 | 0.60 | 0.52 | 1.43 | 1.28 | 0.77 | 0.72 | 2.17 | 1.92 |
| *Genotype P-value* | *0.112* | | *0.304* | | *0.142* | | *0.054* | | ***0.017 (0.04)*** | | *0.099* | |
| *PCB P-value* | *0.999* | | *0.875* | | *0.600* | | *0.469* | | *0.248* | | *0.435* | |
| *Genotype x PCB P-value* | *0.312* | | *0.129* | | *0.919* | | *0.777* | | *0.781* | | *0.720* | |

**Table D.** Wheat biological testing results as means of traits and significance values in Phase II and Phase III testing with significant results highlighted in **bold (**along with average least significant difference given in parentheses).

| PCB | Phase | Seed number | Weight (g) | TGW | Seed area (mm) | Width (mm) | Length (mm) | Germination % |
| --- | --- | --- | --- | --- | --- | --- | --- | --- |
| A | II | 18.85 | 0.46 | 41.03 | 16.85 | 3.48 | 6.14 | 97.41 |
| B | II | 18.15 | 0.46 | 42.49 | 17.53 | 3.56 | 6.20 | 91.33 |
| C1 | II | 21.40 | 0.54 | 41.20 | 17.16 | 3.48 | 6.11 | 91.50 |
| N | II | 19.25 | 0.42 | 33.95 | 15.53 | 3.33 | 5.94 | 82.08 |
| *P-value* | | *0.625* | *0.636* | ***0.020 (5.86)*** | *0.119* | *0.191* | *0.259* | *0.093* |
| C2 | III | 20.76 | 0.91 | 42.21 | 19.10 | 3.66 | 6.64 | 44.46 |
| D | III | 18.80 | 0.83 | 40.06 | 19.45 | 3.54 | 6.89 | 82.04 |
| E | III | 18.48 | 0.68 | 38.76 | 17.58 | 3.41 | 6.43 | 68.55 |
| N | III | 22.19 | 0.71 | 31.66 | 15.17 | 3.24 | 5.79 | 67.76 |
| *P-value* | | *0.528* | *0.255* | ***0.023 (7.08)*** | ***<0.001(2.63)*** | ***0.002 (0.41)*** | ***<0.001 (0.74)*** | *0.059* |

**Table E.** Sugar beet biological testing results as means of traits and significance values in Phase III testing with significant results highlighted in **bold** **(**along with average least significant difference given in parentheses).

| . | Total seed weight (g) | | | | | | | | Number of seeds | | | | | | | |
| --- | --- | --- | --- | --- | --- | --- | --- | --- | --- | --- | --- | --- | --- | --- | --- | --- |
| Genotype | 1 | 2 | 3 | 4 | 5 | 6 | 7 | 8 | 1 | 2 | 3 | 4 | 5 | 6 | 7 | 8 |
| C2 | 6.6 | 7.4 | 10.3 | 5.0 | 14.5 | 10.7 | 8.7 | 6.8 | 704 | 800 | 1026 | 696 | 886 | 1091 | 688 | 997 |
| D | 5.0 | 3.3 | 8.9 | 7.8 | 8.5 | 6.3 | 4.0 | 2.3 | 602 | 464 | 813 | 969 | 604 | 732 | 315 | 385 |
| E | 8.4 | 7.0 | 6.5 | 13.4 | 7.6 | 10.5 | 8.7 | 7.2 | 790 | 873 | 677 | 1462 | 508 | 1287 | 739 | 908 |
| K | 9.5 | 6.0 | 11.5 | 5.3 | 9.8 | 19.4 | 10.2 | 6.7 | 986 | 719 | 1163 | 664 | 643 | 1266 | 786 | 924 |
| Unbagged | 17.4 | 14.3 | 20.9 | 16.3 | 41.2 | 56.9 | 48.8 | 17.6 | 1658 | 1644 | 1828 | 1779 | 2607 | 2770 | 1992 | 2206 |
| *P-value genotype* | ***<0.001 (2.3)*** | | | | | | | | ***<0.001 (213.9)*** | | | | | | | |
| *P-value PCB* | ***<0.001 (1.8)*** | | | | | | | | ***<0.001 (169.1)*** | | | | | | | |
| *P-value gen x PCB* | 0.197 | | | | | | | | ***<0.001 (478.2)*** | | | | | | | |

|  | Germination % | | | | | | | | Number of seedlings | | | | | | | |
| --- | --- | --- | --- | --- | --- | --- | --- | --- | --- | --- | --- | --- | --- | --- | --- | --- |
| Genotype | 1 | 2 | 3 | 4 | 5 | 6 | 7 | 8 | 1 | 2 | 3 | 4 | 5 | 6 | 7 | 8 |
| C2 | 80 | 96 | 100 | 100 | 29 | 90 | 97 | 52 | 60980 | 77837 | 102620 | 69560 | 24913 | 99223 | 67277 | 56231 |
| D | 95 | 100 | 77 | 93 | 32 | 84 | 89 | 51 | 57789 | 46380 | 99608 | 87247 | 18556 | 61397 | 28643 | 21499 |
| E | 100 | 91 | 100 | 100 | 67 | 79 | 87 | 73 | 78975 | 80620 | 67660 | 146180 | 33997 | 126300 | 63533 | 66000 |
| K | 100 | 82 | 100 | 100 | 45 | 97 | 100 | 66 | 98580 | 58318 | 116280 | 66360 | 28421 | 124463 | 78600 | 57998 |
| Unbagged | 20 | 13 | 20 | 17 | 11 | 20 | 20 | 4 | 52960 | 8987 | 53520 | 22183 | 12748 | 64920 | 34800 | 9764 |
| *P-value genotype* | *0.008* | | | | | | | | ***<0.001 (26755)*** | | | | | | | |
| *P-value PCB* | ***<0.001 (10.2)*** | | | | | | | | ***<0.001 (21145)*** | | | | | | | |
| *P-value gen x PCB* | ***<0.001 (28.9)*** | | | | | | | | ***0.745 (59818)*** | | | | | | | |

**Table F.** Internal PCB temperatures recorded using iButton sensors enclosed in test PCBs over both experimental testing phases (Phase II: 4096 observations; Phase III: 4077 observations).

|  |  | Temperature | | | | |
| --- | --- | --- | --- | --- | --- | --- |
| PCB | Phase | Min (°C) | Max (°C) | Range (°C) | Lower quartile | Upper quartile |
| A | II | 14.36 | 41.09 | 26.73 | 18.12 | 27.87 |
| B | II | 14.35 | 40.10 | 25.75 | 18.11 | 26.11 |
| C1 | II | 14.56 | 41.80 | 27.24 | 18.57 | 27.82 |
| N | II | 15.05 | 42.54 | 27.49 | 19.06 | 29.06 |
| Unbagged | II | 14.64 | 37.88 | 23.24 | 18.65 | 25.40 |
| C2 | III | 14.10 | 41.59 | 27.49 | 18.11 | 26.36 |
| D | III | 14.62 | 37.37 | 22.75 | 18.62 | 25.88 |
| E | III | 14.60 | 40.34 | 25.74 | 18.60 | 26.86 |
| N | III | 14.09 | 43.57 | 29.48 | 18.10 | 27.86 |
| Unbagged | III | 15.05 | 42.54 | 27.49 | 19.06 | 29.06 |

**Table G.** Unbagged performance of Phase III sugar beet genotypes for all biological traits.

| Genotype | Total seed weight (g) | Number of seeds | Germination % | Number of seedlings |
| --- | --- | --- | --- | --- |
| 1 | 17.38 | 1658 | 20.00 | 52960.00 |
| 2 | 14.32 | 1644 | 13.33 | 8986.67 |
| 3 | 20.94 | 1828 | 20.00 | 53520.00 |
| 4 | 16.33 | 1779 | 16.67 | 22183.33 |
| 5 | 41.21 | 2607 | 10.93 | 12748.27 |
| 6 | 56.89 | 2770 | 20.00 | 64920.00 |
| 7 | 48.75 | 1992 | 20.00 | 34800.00 |
| 8 | 17.62 | 2206 | 4.47 | 9764.13 |

**Table H.** Correlation between physical and biological properties for sugar beet genotypes G_01 and G_98 Phase II testing with significant results highlighted in **bold**. R^2^ correlation values are given in parentheses for significant results.

|  | Total seed weight (g) | | Number of seeds | | Germination % | |
| --- | --- | --- | --- | --- | --- | --- |
| Physical property | G_01 | G_98 | G_01 | G_98 | G_01 | G_98 |
| Thicknesss (mm) | 0.381 | 0.082 | 0.476 | 0.073 | 0.928 | 0.371 |
| Weight (gm^-2^) | 0.655 | 0.958 | 0.752 | 0.927 | 0.693 | 0.276 |
| % transmittance^1^ | 0.161 | **0.015 (0.971)** | 0.172 | **0.015 (0.921)** | 0.548 | 0.206 |
| Air permeability (1m^-2^s^-1^) | 0.713 | 0.251 | 0.789 | 0.228 | 0.844 | 0.262 |
| Max. pore size (um) | 0.482 | 0.115 | 0.504 | 0.098 | 0.767 | 0.090 |
| WVT (%) | 0.503 | 0.127 | 0.522 | 0.110 | 0.767 | 0.083 |

**Table I.** Correlation between physical and biological properties by genotype (denoted 1-8) for sugar beet Phase III testing with significant results highlighted in **bold**. R^2^ correlation values are given in parentheses for significant results.

|  | Total seed weight (g) | | | | | | | |
| --- | --- | --- | --- | --- | --- | --- | --- | --- |
| Physical property | 1 | 2 | 3 | 4 | 5 | 6 | 7 | 8 |
| Thicknesss (mm) | 0.579 | **0.028 (0.944)** | 0.899 | 0.886 | 0.335 | 0.676 | 0.234 | 0.127 |
| Weight (gm^-2^) | 0.891 | 0.422 | 0.524 | 0.379 | 0.010 | 0.913 | 0.650 | 0.618 |
| % transmittance^1^ | 0.059 | 0.280 | 0.637 | 0.856 | 0.824 | 0.086 | **0.018 (0.964)** | 0.121 |
| Air permeability (1m^-2^s^-1^) | 0.588 | 0.729 | 0.053 | 0.146 | 0.403 | 0.218 | 0.416 | 0.656 |
| Max. pore size (um) | 0.586 | **0.041 (0.920)** | 0.837 | 0.823 | 0.298 | 0.651 | 0.231 | 0.139 |
| WVT (%) | 0.412 | 0.751 | 0.455 | 0.708 | 0.660 | 0.217 | 0.682 | 0.970 |
|  | Number of seeds | | | | | | | |
| Thicknesss (mm) | 0.698 | 0.124 | 0.726 | 0.869 | 0.427 | 0.322 | 0.219 | 0.068 |
| Weight (gm-2) | 0.922 | 0.656 | 0.478 | 0.400 | **0.031 (0.939)** | 0.903 | 0.704 | 0.450 |
| % transmittance1 | 0.070 | 0.281 | 0.447 | 0.782 | 0.908 | 0.076 | **0.041 (0.920)** | 0.134 |
| Air permeability (1m-2s-1) | 0.389 | 0.909 | **0.006 (0.989)** | 0.107 | 0.404 | 0.702 | 0.538 | 0.528 |
| Max. pore size (um) | 0.687 | 0.151 | 0.669 | 0.806 | 0.385 | 0.339 | 0.225 | 0.070 |
| WVT (%) | 0.234 | 0.793 | 0.441 | 0.642 | 0.676 | 0.737 | 0.758 | 0.997 |
|  | Germination % | | | | | | | |
| Thicknesss (mm) | 0.118 | 0.725 | 0.118 | 0.118 | 0.876 | 0.533 | 0.746 | 0.814 |
| Weight (gm-2) | 0.570 | 0.887 | 0.570 | 0.570 | 0.446 | 0.816 | 0.432 | 0.498 |
| % transmittance1 | 0.098 | 0.081 | 0.098 | 0.098 | 0.626 | 0.162 | 0.532 | 0.445 |
| Air permeability (1m-2s-1) | 0.574 | 0.411 | 0.574 | 0.574 | 0.536 | 0.884 | **0.019 (0.999)** | 0.740 |
| Max. pore size (um) | 0.125 | 0.716 | 0.125 | 0.125 | 0.929 | 0.560 | 0.687 | 0.859 |
| WVT (%) | 0.931 | 0.223 | 0.931 | 0.931 | 0.924 | 0.655 | 0.505 | 0.750 |
|  | Number of seedlings | | | | | | | |
| Thicknesss (mm) | 0.862 | 0.118 | 0.897 | 0.984 | 0.443 | 0.377 | 0.233 | 0.180 |
| Weight (gm-2) | 0.716 | 0.553 | 0.641 | 0.458 | 0.878 | 0.971 | 0.607 | 0.733 |
| % transmittance1 | 0.151 | 0.540 | 0.819 | 0.900 | 0.267 | 0.080 | **0.019 (0.409)** | 0.141 |
| Air permeability (1m-2s-1) | 0.530 | 0.899 | 0.142 | 0.166 | 0.940 | 0.720 | 0.360 | 0.759 |
| Max. pore size (um) | 0.859 | 0.149 | 0.960 | 0.920 | 0.480 | 0.395 | 0.225 | 0.200 |
| WVT (%) | 0.183 | 0.477 | 0.443 | 0.653 | 0.907 | 0.688 | 0.665 | 0.966 |
